# Heritable Risk and Protective Genetic Components of Glaucoma Medication Non-Adherence

**DOI:** 10.3390/ijms24065636

**Published:** 2023-03-15

**Authors:** Julie L. Barr, Michael Feehan, Casey Tak, Leah A. Owen, Robert C. Finley, Parker A. Cromwell, John H. Lillvis, Patrice M. Hicks, Elizabeth Au, Michael H. Farkas, Asher Weiner, Andrew L. Reynolds, Sandra F. Sieminski, Richard M. Sherva, Mark A. Munger, Murray H. Brilliant, Margaret M. DeAngelis

**Affiliations:** 1 Neuroscience Graduate Program, Jacobs School of Medicine and Biomedical Sciences, State University of New York, University at Buffalo, Buffalo, NY 14203, USA; 2 Department of Ophthalmology, Ross Eye Institute, Jacobs School of Medicine and Biomedical Sciences, State University of New York, University at Buffalo, Buffalo, NY 14203, USA; 3 Cerner Enviza, an Oracle Company, Kansas City, MO 64117, USA; 4 Departments of Pharmacotherapy and Internal Medicine, University of Utah Health, Salt Lake City, UT 8413, USA; 5 Department of Ophthalmology and Visual Sciences, University of Utah School of Medicine, The University of Utah, Salt Lake City, UT 84132, USA; 6 Department of Population Health Sciences, University of Utah School of Medicine, The University of Utah, Salt Lake City, UT 84132, USA; 7 Department of Obstetrics and Gynecology, University of Utah School of Medicine, The University of Utah, Salt Lake City, UT 84132, USA; 8 Veterans Administration Western New York Healthcare System, Buffalo, NY 14212, USA; 9 Department of Ophthalmology and Visual Sciences, University of Michigan, Ann Arbor, MI 48105, USA; 10 Department of Medicine (Biomedical Genetics), Boston University Chobanian & Avedisian School of Medicine, Boston, MA 02118, USA; 11 Center for Precision Medicine Research, Marshfield Clinic Research Institute, Marshfield, WI 54449, USA; 12 Department of Biochemistry, Jacobs School of Medicine and Biomedical Sciences, State University of New York, University at Buffalo, Buffalo, NY 14203, USA; 13 Genetics, Genomics and Bioinformatics Graduate Program, Jacobs School of Medicine and Biomedical Sciences, State University of New York, University at Buffalo, Buffalo, NY 14203, USA

**Keywords:** glaucoma, medication, adherence, non-adherence, genetic, heritability, protection, risk

## Abstract

Glaucoma is the leading cause of irreversible blindness, affecting 76 million globally. It is characterized by irreversible damage to the optic nerve. Pharmacotherapy manages intraocular pressure (IOP) and slows disease progression. However, non-adherence to glaucoma medications remains problematic, with 41–71% of patients being non-adherent to their prescribed medication. Despite substantial investment in research, clinical effort, and patient education protocols, non-adherence remains high. Therefore, we aimed to determine if there is a substantive genetic component behind patients’ glaucoma medication non-adherence. We assessed glaucoma medication non-adherence with prescription refill data from the Marshfield Clinic Healthcare System’s pharmacy dispensing database. Two standard measures were calculated: the medication possession ratio (MPR) and the proportion of days covered (PDC). Non-adherence on each metric was defined as less than 80% medication coverage over 12 months. Genotyping was done using the Illumina HumanCoreExome BeadChip in addition to exome sequencing on the 230 patients (1) to calculate the heritability of glaucoma medication non-adherence and (2) to identify SNPs and/or coding variants in genes associated with medication non-adherence. Ingenuity pathway analysis (IPA) was utilized to derive biological meaning from any significant genes in aggregate. Over 12 months, 59% of patients were found to be non-adherent as measured by the MPR80, and 67% were non-adherent as measured by the PDC80. Genome-wide complex trait analysis (GCTA) suggested that 57% (MPR80) and 48% (PDC80) of glaucoma medication non-adherence could be attributed to a genetic component. Missense mutations in *TTC28*, *KIAA1731*, *ADAMTS5*, *OR2W3*, *OR10A6*, *SAXO2*, *KCTD18*, *CHCHD6*, and *UPK1A* were all found to be significantly associated with glaucoma medication non-adherence by whole exome sequencing after Bonferroni correction (*p* < 10^−3^) (PDC80). While missense mutations in *TINAG*, *CHCHD6*, *GSTZ1*, and *SEMA4G* were found to be significantly associated with medication non-adherence by whole exome sequencing after Bonferroni correction (*p* < 10^−3^) (MPR80). The same coding SNP in *CHCHD6* which functions in Alzheimer’s disease pathophysiology was significant by both measures and increased risk for glaucoma medication non-adherence by three-fold (95% CI, 1.62–5.8). Although our study was underpowered for genome-wide significance, SNP rs6474264 within ZMAT4 (*p =* 5.54 × 10^–6^) was found to be nominally significant, with a decreased risk for glaucoma medication non-adherence (OR, 0.22; 95% CI, 0.11–0.42)). IPA demonstrated significant overlap, utilizing, both standard measures including opioid signaling, drug metabolism, and synaptogenesis signaling. CREB signaling in neurons (which is associated with enhancing the baseline firing rate for the formation of long-term potentiation in nerve fibers) was shown to have *protective* associations. Our results suggest a substantial heritable genetic component to glaucoma medication non-adherence (47–58%). This finding is in line with genetic studies of other conditions with a psychiatric component (e.g., post-traumatic stress disorder (PTSD) or alcohol dependence). Our findings suggest both risk and protective statistically significant genes/pathways underlying glaucoma medication non-adherence for the first time. Further studies investigating more diverse populations with larger sample sizes are needed to validate these findings.

## 1. Introduction

Glaucoma is a chronic, neurodegenerative, and progressive condition and the most common cause of blindness globally [1,2,3]. It is characterized by irreversible damage to the optic nerve and attenuation of the retinal cell fiber layer leading to blindness with currently more than 76 million people afflicted worldwide [4,5]. The prevalence of glaucoma is higher in Black and Hispanic populations compared to non-Hispanic-White populations [6,7,8].

Although there is no cure for glaucoma, it can be managed via the reduction of intraocular pressure (IOP) through the effective use of medications and surgeries [1,9,10]. Early diagnosis and treatment can control glaucoma before vision loss occurs. As vision loss becomes advanced and surgery is required, most of those patients receive one or more medications and are advised to use eye drops daily and indefinitely.

However, patient adherence to glaucoma medication is problematic with non-adherence being shown to significantly contribute to disease progression and avoidable vision loss in patients [11,12]. Despite substantial investment in research, clinical efforts, and patient education protocols, adherence to glaucoma medication remains low, ranging from 30% to 37% after one year [11,12,13,14,15,16]. Medication non-adherence rates are similar to oral medications for other chronic asymptomatic conditions such as hypertension or hypercholesterolemia [17]. Adherence to medication can have numerous barriers, including adverse medication side effects, lack of accessibility to a healthcare provider, health literacy, memory failure, cost of prescriptions, and patients’ awareness of vision loss risk [13,14,15,16]. In addition to the per-patient health benefit, recent studies have shown that the cost utility of non-adherence to glaucoma medication is around $29,000/quality-adjusted life year making it highly cost-effective if medication non-adherence can be improved [18].

Even when clinical and educational barriers to satisfactory glaucoma medication adherence are reduced, continued non-adherence persists. Therefore, non-adherence could potentially have an underlying biological and genetic component. A genetic component for medication non-adherence could aid in the partial explanation for non-adherence in a condition in which patients know that non-adherence increases the likelihood of blindness [19,20]. Identification of these genetic markers could conceivably facilitate screening for more at-risk patients for non-adherence, and the development of focused treatment plans could improve adherence rates among those so predisposed. Recent studies have been conducted on the genetics of medication non-adherence in some chronic conditions such as diabetes and hypertension [21]. In the management of high cholesterol with statin therapy, several genetic variants involved in the modulation of statin absorption and drug metabolizing enzymes of statins have been shown to affect rates of medication adherence [21]. An additional cause of non-adherence that can be seen with chronic medications is adverse side effects to the medication (e.g., statin-associated musculoskeletal symptoms), although few severe adverse side effects have been reported with glaucoma medications [22,23,24]. Patients may be less likely to adhere when the perception of long-term consequences of non-adherence medication are outweighed by concerns when current adverse side effects are experienced [24]. A study investigating medication adherence in diabetes and hypertension was conducted with the Korean Association Resource (KARE) [22]. This genome-wide association study (GWAS) of 1,032 female patients identified single nucleotide polymorphisms (SNPs) that were significantly associated with patient self-reported non-adherence [22]. Although no SNPs reached genome wide significance, one SNP, proximal to *GCC1*, was found to be nominally significant (10^−6^). Interestingly, *GCC1* is implicated in the decision-making of people with substance use disorder [22]. The involvement of genes in both decision making and drug metabolism processes supports the need to better understand the genetics behind medication non-adherence.

The present study aimed to (1) determine levels of glaucoma medication non-adherence using two standardized metrics to interrogate pharmacy refill data in the Marshfield Clinic Healthcare System, (2) determine the heritability of glaucoma medication non-adherence, and (3) determine if genetic markers are associated with glaucoma medication non-adherence. 

## 2. Results

### 2.1. Non-Adherence Proportions 

The prevalence of non-adherence was high, with 66.5% identified as non-adherent using the PDC80 and 58.7% non-adherence using the MPR80 (Table 1). Of the 230 patients with genetic data, 137 (59%) were non-adherent for both measures. Non-adherence was higher in men in both measures, with 72% (PDC80) and 64% (MPR80), while non-adherence seen in women was 62% (PDC80) and 56% (MPR80) (Table 1). The age range of patients in our study was 43–99 with an average age of 78, and 38% of the study population were male. When comparing 40–64-year-olds with people 65 and over, the 40–64 group had higher levels of non-adherence for both measures (Table 1). Race was not examined individually as all of the patients with genetic profiles were White Non-Hispanic.

### 2.2. Sample Size Calculation

Utilizing the standard measure PDC80 and a disease prevalence of 67% as represented in our cohort (n = 230), the calculated power was 59% (*p* = 10^−6^). For the standard measure MPR80, a disease prevalence of 59% was used as seen in our population (n = 230) demonstrate power of 67% to detect statistical significance at a genome wide level. Although previous studies have shown genome-wide significance at a *p*-value of 10^−6^, due to the underpowered nature of this study, a *p*-value of 10^−6^ is classified as nominal genome-wide significance. 

### 2.3. Genotyping and Exome Sequencing

Under an additive model, single SNP analysis was performed controlling for the age, sex, and the first four principal components. This analysis determined that there were 13,341 and 13,588 SNPs with a *p* < 0.05 for PDC80 and MPR80, respectively, with 7258 and 7308 of these SNPs identified through both whole exome sequencing and the Illumina chip in PDC80 and MPR80, respectively. One SNP, rs6474264, in *ZMAT4* reached nominal genome wide-significance, with *p* = 5.54 × 10^−6^ (OR, 0.22, (CI, 0.11–0.42) in PDC80 with the same SNP observed in MPR80 with a *p*-value of 1.3 × 10^−4^ (OR 0.29 (CI, 0.15–0.54) (Table 2). This SNP is located in intron 5 of *ZMAT4* and is 13.1 kb from exon 5. In PDC80, 11 additional SNPs in 11 loci were found to be nominally significant (*p* < 10^−5^), and eight SNPs were identified as nominally significant in MPR80 (Table 2). One SNP, rs7571026, in lINC01804 was found to be nominally significant by both PDC80 and MPR80 (*p* < 10^−5^) (OR, 0.4: CI, 0.25–0.6). It is interesting to note that both of these changes are protective against medication non-adherence (Table 2).

Of most significant coding variants identified from exome sequencing by PDC80 following Bonferroni correction (Table 3), SNPs rs9613558 (*TTC28*), rs7131178 (*KIAA1731*), and rs4758258 (*OR10A6*) were all found to be associated with protection or lower risk of glaucoma medication non-adherence (Table 3). Of the most significant coding variants identified by MPR80 following Bonferroni correction (Table 3), only one SNP, rs3195579 (*TINAG*), was found to be associated with protection or lower risk of glaucoma medication non-adherence (Table 3). Of the most significant coding variants associated with increased risk of glaucoma medication non-adherence, following Bonferroni correction (Table 3), SNPs rs2830585 (*ADAMTS5*), rs10888267 (*OR2W3*), rs16973457 (*SAXO2*), rs3795969 (*KCTD18*), rs2272487 (*CHCHD6*), and rs6741212 (*UPK1A*) were identified by the more conservative measure PDC80 (Table 3). Of most significant coding variants associated with increased risk of glaucoma medication non-adherence (Table 3), rs2272487 (*CHCHD6*), rs7975 (*GSTZ1*), and rs11591349 (*SEMA4G*) were identified by MPR80 (Table 3). Only one coding SNP, rs2272487, located in exon 4 and identified from the exome sequencing data, in the *CHCHD6* gene, was identified with an increased risk of glaucoma medication adherence. All coding SNPs that resulted in amino acid changes resulted in missense mutations.

### 2.4. Heritability

GCTA was utilized to perform GREML analysis to test for the heritability of genetic components associated with non-adherence. The genetic component of glaucoma medication non-adherence was 57.6% for MPR80 and 48.14% for PDC80 (Table 4). Although these results are not significant (MPR80 *p* = 0.17, PDC80 *p* = 0.20), the heritable component is substantial, which may be indicative of sample size, limiting the determination of statistical significance.

### 2.5. Bioinformatics

IPA analysis of canonical pathways from identified SNPs with a cutoff of *p* < 0.05 was performed for both MRP80 and PDC80, separately and together. A total of 207 significant canonical pathways were identified for the PDC80, while 156 significant pathways were identified for the MPR80. There was a 60% overlap of the ten most significant pathways between each measure (Table 5). Overall, there were 134 significant pathways that were identified in both PDC80 and MPR80, with a total overlap of 86% in MPR80 and 65% in PDC80.

Then, genes significant for protection of non-adherence and separately risk of non-adherence to glaucoma medication were analyzed by both measures separately to identify pathways unique to either risk or protection. A protection:risk ratio (P:R) was developed to show the proportion of genes in a pathway that were associated with protection or risk. A P:R ratio greater than 1 indicates that there are more protective genes in a pathway, while a P:R ratio less than one indicated that there are more risk genes in a pathway. Pathways with a P:R ratio less than 0.5 or greater than 1.5 were used as a threshold to label a pathway overall as protective or risk [25]. The p-values listed are the significance of the pathway within either PDC80 or MPR80.

Protection: The pathway in PDC80 with the highest P:R ratio was the CREB signaling in neurons pathway, with a P:R of 1.38. The CREB signaling in neurons pathway was also examined for the MPR80 and was significantly associated with protection from non-adherence on this measure (*p* = 3.37 × 10^−4^, P:R 2.05). A total of 14 protective pathways were seen in MPR80 with a P:R of 1.5 or higher: the opioid signaling pathway (*p* = 3.7 × 10^−8^), the neuropathic pain signaling in dorsal horn neurons pathway (*p* = 8.12 × 10^−8^), the semaphorin neuronal repulsive signaling pathway (*p* = 1.16 × 10^−7^), the role of NFAT in cardiac hypertrophy pathway (*p* = 4.76 × 10^−7^), the cardiac β-adrenergic signaling pathway (*p* = 1.09 × 10^−6^), the cAMP-mediated signaling pathway (*p* = 2.24 × 10^−6^), the synaptic long term potentiation pathway (*p* = 3.64 × 10^−6^), the Fcγ receptor-mediated phagocytosis in macrophages and monocytes pathway (*p* = 9.88 × 10^−6^), the cellular effects of sildenafil pathway (*p* = 2.13 × 10^−5^), the sperm motility pathway (*p* = 2.37 × 10^−5^), the GPCR-mediated nutrient sensing in enteroendocrine cells pathway (*p* = 2.94 × 10^−5^), the endocannabinoid neuronal synapse pathway (*p* = 4.73 × 10^−5^), the melatonin signaling pathway (*p* = 4.95 × 10^−5^), and the white adipose tissue browning pathway (*p* = 6.77 × 10^−5^). The pathway in PDC80 with the highest P:R ratio was the CREB signaling in neurons (*p* = 1.87 × 10^−6^), with a P:R of 1.38. The CREB signaling in neurons pathway was also examined for the MPR80 and was significantly associated with protection from non-adherence on this measure (*p* = 3.37 × 10^−4^, P:R 2.05).

Risk: The long-term potentiation pathway was seen in both measures but shown as a risk feature for PDC80 and a protective feature for MPR80. For non-adherence as measured by the PDC80, four pathways (the synaptic long-term potentiation pathway (*p*= 3.21 × 10^−9^), the paxillin signaling pathway (*p* = 4.43 × 10^−7^), the thrombin signaling pathway (*p* = 9.70 × 10^−7^), and the integrin signaling pathway (*p* = 1.13 × 10^−6^)) were identified with a protection:risk (P:R) ratio of 0.5, showing a potential for risk association. For non-adherence measured with the MPR80, no pathways had a P:R ratio of 0.5, but the extracellular signal regulated kinases (ERK)/mitogen-activated protein kinases (MAPK) signaling pathway (*p* = 2.83 × 10^−5^, P:R, 0.87), which has roles in the regulation of cellular proliferation, and the xenobiotic metabolism signaling pathway (*p* = 3.59 × 10^−5^, P:R, 0.89), which is involved in the breakdown of drugs, had the lowest P:R ratios [26,27,28].

IPA analysis of the intronic and coding SNPs separately showed a total of 335 significant pathways in PDC80 intronic SNPs with the top three most significant pathways identified being the opioid signaling pathway, the endocannabinoid cancer inhibition pathway, and the corticotropin releasing hormone signaling pathway, while only one significant pathway was seen with the PDC80 coding SNPs, the role of chondrocytes in rheumatoid arthritis signaling pathway (Table S1). In MPR80, there were seven significant pathways identified from the intronic SNPs with the top three most significant pathways being the TR/RXR activation pathway, the non-small cell lung cancer signaling pathway, and the small cell lung cancer signaling pathway (Table S1). Thirteen significant pathways were identified for MPR80 coding SNPs, with the top three being the tyrosine degradation I pathway, the glutathione redox reactions I pathway, and the glutathione-mediated detoxification pathway (Table S1). When conduction pathway analysis on all of the coding SNPs in both MPR80 and PDC80, five significant pathways were identified: the tyrosine degradation I pathway, the glutathione redox reactions I pathway, the glutathione-mediated detoxification pathway, the xenobiotic metabolism AHR signaling pathway, and the apelin adipocyte signaling pathway (Table S1).

## 3. Discussion

Our study is the first to identify genetic associations that can be attributed to glaucoma medication non-adherence through two well-established dispensing-based measures (PDC80 and MPR80), with both thresholds for unsatisfactory adherence set at <80%. Previous research has shown the utility of these measures to determine the prevalence of glaucoma medication adherence [17]. In our study, the prevalence of non-adherence was high, with an overall non-adherence of 66.7% in PDC80 and 58.4% in MPR80 (Table 1). The prevalence of non-adherence in this study is in line with our previous findings [17].

Further analysis showed that the heritability of glaucoma medication non-adherence was high (between 57% and 48%), which is comparable to the genetic heritability of psychological conditions including post-traumatic stress disorder (PTSD), alcohol dependence, and smoking behaviors and lends some validity to our estimate of glaucoma medication non-adherence heritability (Table 4) [29,30,31]. While there was a substantial genetic variance between adherent and non-adherent groups, the significance is limited due to the small sample size as clearly demonstrated by the power calculations that show we do not have 80% to detect genome wide significance. Specifically, utilizing two well-established dispensing-based measures (PDC80 and MPR80) we found that our sample size had 59–67% power to detect statistical significance at the genome-wide level. However, the whole exome sequencing approach on all 230 samples identified 13 unique coding variants in 13 genes, all significant after Bonferroni correction (10^−3^).

In addition to the high level of genetic heritability shown, canonical pathway analysis identified potential biological mechanisms that may be associated with glaucoma medication non-adherence. Identification of protective and risk genes/pathways involved in non-adherence can potentially allow for development of risk-based screening and more targeted interventions that could decrease the rates of non-adherence. Of interest, the CREB signaling in neurons pathway was identified as a protective pathway by both measures. The CREB pathway has been found to increase neuronal excitability and in turn enhance long-term synaptic plasticity through long-lasting changes in memory and circuit structure [32,33,34]. This could play a key role in the reduction of medication non-adherence as an increase in long-term memory circuits could decrease the impact of memory challenges to maintaining satisfactory adherence.

Genes associated with the coding SNPs as measured by PDC80 and MPR80 have been previously attributed to cognitive functions including smoking initiation (*OR10A6*) and detoxification of drugs (*GST1*) [29,35,36]. The coding SNP rs2272487 in *CHCHD6* that overlapped as measured by both MPR80 and PDC80 groups was identified as having a proportionally higher OR for glaucoma non-adherence. Due to the *CHCHD6* being a mitochondrial gene, there is potential that there may be significant impact of mitochondrial genes or metabolic pathways that are associated with medication non-adherence [35]. Previous studied have implicated the role of *CHCHD6* in neurodegenerative diseases such as Alzheimer’s disease [36]. Though few studies have been conducted on the function of *ZMAT4*, it has been implicated in myopia as well as thyroid cancer [37,38]. rs6474264, identified in intron 5 of *ZMAT4* as being nominally significant by both measures, is protective of glaucoma medication non-adherence. Of note, rs7829127 and rs2137277, located in intron 1 of *ZMAT4*, were found to be associated by both GWAS and meta-analysis with the risk of high myopia in the Han-Chinese [37,38,39].

Additionally, the presence of opioid signaling and xenobiotic metabolism signaling introduces another potential layer that may underpin the role of drug breakdown and efficacy in glaucoma medication non-adherence. Xenobiotics are chemical substances that can be either endogenous or exogenous, including drugs, environmental pollutants, and industrial chemicals [40,41,42]. If there is an alteration of the rate of medication metabolism associated with medication non-adherence, altering the chemical makeup of the administered drug may allow for lower rates of non-adherence as they would not be broken down as swiftly [40,41,42]. Many of these significant coding SNPs are in pathways involved in xenobiotic drug metabolism as well as glutathione mediated detoxification, which has been implicated in drug breakdown, suggesting that an aspect of the genetic component to medication non-adherence could be due to an alteration in drug metabolism and excretion [43]. Identification of the xenobiotic metabolism and glutathione metabolism in both genes with coding and non-coding SNPs with p values that were significantly below *p* < 0.05 shows the importance of these pathways in glaucoma medication non-adherence.

Replication of this study using a larger and more diverse population would allow for the validation of these findings. As the prevalence of glaucoma is much higher in Black and Hispanic individuals, a more diverse study is necessary, as the Marshfield population is predominantly White, non-Hispanic [6,8,44]. In a larger replication sample size with greater statistical power, multivariate statistical modeling could examine the intercorrelations of the two non-adherence metrics and adjust for potentially confounding demographic and other patient or treatment characteristics (e.g., visual impairment progression and co-morbid conditions). Such modeling may provide a clearer picture of the role of genetics in medication non-adherence. Moreover, future research in a larger, more diverse population may allow for explorations of gene–environment interactions with glaucoma medication non-adherence.

In summary, this study established the high prevalence of glaucoma medication non-adherence through medical records, identified substantial heritable genetic components, and began to explore genetic protective and risk factors for glaucoma medication non-adherence and possible associated biological pathways for mechanisms of action. Identification of biological factors for non-adherence may provide objective measures that identify individuals who may need additional support to maintain their chronic medication adherence. Genetic non-adherence patterns and pathways may provide clues for drug therapy development that could enhance adherence and treatment of glaucoma and other chronic conditions if so replicated. Our results provide some insight into the underlying biological mechanisms of medication non-adherence that, if validated with larger and more diverse study populations, has the potential to inform clinical screening and intervention and potentially even drug development, which could positively impact the levels of non-adherence seen in glaucoma drug development and which could positively impact the levels of non-adherence seen in glaucoma.

## 4. Methods

The protocol was reviewed and approved by the institutional review board at the University at Utah (IRB 52879) and conforms to the tenets of the Declaration of Helsinki.

### 4.1. Data source and Study Sample

The Marshfield Clinic Healthcare System serves a patient population in more than 50 locations throughout northern, central, and western Wisconsin. The Marshfield Clinic’s pharmacy dispensing database was used to identify the records of patients’ receiving prescriptions for glaucoma medications. Data was abstracted from 279 participants in The Personalized Medicine Research Project as previously described [45]. These participants had 365 days of potential glaucoma medication coverage, with 230 of these participants having genetic data paired with their electronic health records.

### 4.2. Calculation of Non-Adherence

A multi-measure approach to phenotype non-adherence from one calendar year of dispensing data in the patients’ electronic medical records (VINCI) leveraged two established metrics of adherence: the proportion of days covered (PDC) and the medication possession ratio (MPR) over 12 months. MPR is calculated by dividing the number of days of supply of medicine by the number of days from the first dispensing date to the end of the duration of the last refill within the 12-month exposure period. PDC represents the number of days that a patient had the medication over the observed time period. MPR can overestimate adherence, for example through patients re-filling their prescriptions early and having more supply on hand. The PDC is seen as more conservative as it focuses on the number of days covered. In the above example of a patient filling a prescription early, the PDC would be lower than the MPR as a day with extra supply would still be treated as only one 1 covered day [46].

For both measures, non-adherence is defined as MPR or PDC less than 80% of supply available to the patient. The cutoff of 80% has been used in prior studies of glaucoma medication non-adherence, and both the PDC80 and MPR80 cutoffs have been used previously by the authors in prior database analyses for glaucoma and asthma non-adherence [17,47]. Control groups were defined for both PDC80 and MPR80 separately, with control individuals defined as adherent to their medication at least 80% of the time over the 1-year period.

### 4.3. Glaucoma Medications

Patients with a diagnosis of glaucoma (ICD9-code) who were prescribed appropriate medications were identified between May 2013 and May 2015. At the class level, medications included in this study were prostaglandins, rho kinase inhibitors, nitric oxides, miotic or cholinergic agents, alpha-adrenergic agonists, beta blockers, and carbonic anhydrase inhibitors. This period allowed for the ascertainment of 1 full year of potential medication coverage for each patient. Insurance key methods were used to capture glaucoma medication refills made from outside the Marshfield Clinic Healthcare System in the electronic record, allowing greater confidence that the target subset population was truly non-adherent.

### 4.4. Genotyping, Whole Exome Sequencing, and Calculation of Sample Size

DNA samples were genotyped using the Illumina HumanCoreExome BeadChip v1.1 (2016) (569,645 variants) and exome sequencing (Illumina Inc., San Diego, CA, USA). SNP data cleaning and analysis were performed using PLINK v1.07 (http://pngu.mgh.harvard.edu/~purcell/plink/) (accessed on 24 July 2017) [48]. Standard procedures were used to perform data cleaning as previously described [49]. Population sub-structure assessment in this dataset was performed using principal components analysis (PCA) with Eigensoft (http://www.hsph.harvard.edu/alkes-price/software/) (accessed on 24 July 2017) followed by Bonferroni corrections [50,51]. Based on previous studies, the Illumina exome chip, with 569,645 variants, a threshold of *p* < 10^−6^, was proposed to be used for genome-wide significance as the MAF >5% [52]. However, to determine true genome wide significance with our sample size, a power calculation was conducted. Specifically, a power calculation for PDC80 and another separately for MPR80 were conducted to determine the statistical power for this study [53]. In both PDC80 and MPR80, coding and intronic SNPs were identified and examined. All coding SNPs were identified through exome sequencing. The effect of these SNPs on the protein structure in the form of amino acid change and protein function was also examined using both PolyPhen and SIFT through the SNPNexus tool [54,55]. IPA analysis was then conducted separately on the MPR80 and PDC80 coding SNPs and intronic SNPs with one IPA analysis conducted on a list of all of the coding SNPs from both PDC80 and MPR80.

For the SNP analysis, the minor allele for each SNP was tested for association with non-adherence using logistic regression in PLINK. Association tests were performed controlling for age and sex along with the significant principal components (PCs). The dataset was then permuted 10,000 times, keeping linkage disequilibrium between SNPs constant and varying the phenotype labels. The intervals were obtained using index variants and proxies, r^2^ ≥ 0.5 and ±100 kb.

### 4.5. Heritability Calculations

Genome-wide complex trait analysis (GCTA) was used to estimate the heritability of non-adherence [56]. Of the 279 total patients with medication adherence metrics, the 230 with genetic data were used to calculate heritability with data from the exome sequencing and Illumina chip used. There are three main components to heritability: genetics, phenotype, and environment [56,57]. These components use variance among a group of patients to show how much of a trait can be attributed to each component. As the participants in our study were unrelated, there is no shared environment between them, so the environmental component is excluded [57]. Two tests are used to form a genomic-relatedness-based restricted maximum-likelihood (GREML) analysis to determine heritability, the genetic relatedness matrix (GRM) and residual maximum likelihood (REML). First, a GRM is calculated based on if individuals share the same genetic variation at a given locus by comparing SNPs between all 230 patients in the sample. Genetic relatedness allows for the prediction of phenotypic relatedness through the grouping of SNPs across individuals. Due to the unrelated sample of patients in the study, the standard GRM cutoff of 0.025 was used. A REML was then performed to estimate the weight of each genetic, phenotype, and environmental variance components across the medication adherent and medication non-adherent groups.

### 4.6. Bioinformatic Analysis

Ingenuity pathway analysis (IPA) (QIAGEN Inc., 1001 Marshall St, Redwood City CA 94063, United States) (https://digitalinsights.qiagen.com/IPA) was used to determine canonical pathways and networks involved with the genes associated with the exome sequencing and Illumina chip data of both PDC80 and MPR80 [58]. Through the IPA database, predictions can be made of what pathways may be altered based on the list of genes that were identified as associated with non-adherence.

To determine the presence of potentially significant protective and risk genetic factors, the odds ratio (OR) associated with each SNP was used. An OR less than 1 was classified as protective, while higher than 1 was classified as a risk factor. A protection:risk ratio (P:R) was developed based on previous research to show the proportion of genes in a pathway that were associated with protection or risk [25,59]. First, SNPs mapped to a gene were identified as protective (OR < 1) or risk (OR > 1). Next, these protective or risk genes were identified within each significant pathway, and the total number of protective and risk genes were totaled, and the P:R ratio was seen through the number of protective genes in a pathway divided by the number of risk genes in that same pathway. Using the top 30 significant pathways, pathways with a protection:risk ratio (P:R) genes identified with a ratio over 1.5 or under 0.5 were identified.

## Figures and Tables

**Table 1:** Age and sex characteristics of the non-adherence subgroups of all patients with prescription data analyzed in the study (n = 230).

		Sex		Age	
Proportion Non-adherent:	Total (n = 230)	Male (n = 88)	Female (n = 142)	40–64 (n = 19)	65 and Over (n = 211)
Proportion of Days Covered (PDC < 80)	153 (67%)	64 (72%)	89 (62%)	15 (79%)	138 (65%)
Medicine Possession Ratio (MPR < 80)	135 (59%)	56 (64%)	79 (56%)	13 (68%)	122 (58%)

**Table 2:** Significant and Nominally Significant SNPs in PDC80 and MPR80.

	SNP rsID	Position	bp (hg19)	ENSEMBL Name	Gene Name	Ref/ Alt	MAF (%)	OR	95% CI Lower	95% CI Upper	*p*-Value
PDC80	rs6474264	8q21.11	40519227	ENSG00000165061	*ZMAT4*	T/C	13	0.22	0.11	0.42	5.45 × 10^−6^
	rs1496750	4q23	16624209	ENSG00000169744	*LDB2*	C/T	46	0.35	0.22	0.56	1.1 × 10^−5^
	rs2388079	13q21.2	30000621	ENSG00000132938	*MTUS2*	T/C,A	18	0.28	0.16	0.50	1.59 × 10^−5^
	rs8277	10q21.3	125505401	ENSG00000121898	*CPXM2*	A/G	35	0.36	0.22	0.57	1.96 × 10^−5^
	rs203884	6q14.3	28077374	ENSG00000269293.5	*ZNF165/ ZSCAN16-AS1*	C/A	27	3.41	1.92	6.05	2.7 × 10^−5^
	rs9613558	22q11.22	28378688	ENSG00000100154	*TTC28*	C/G	7	0.18	0.08	0.40	3.46 × 10^−5^
	rs149901	6q14.3	27965503	ENSG00000216629	*OR2W4P/ LOC340192*	C/T,A	22	3.59	1.95	6.60	4.18 × 10^−5^
	rs149942	6q14.3	28001610	ENSG00000217315	*OR2W2P*	T/C	27	3.20	1.82	5.61	5.13 × 10^−5^
	rs1150678	6q14.3	28142276	ENSG00000216901	*ZNF603P*	C/T	26	3.19	1.82	5.60	5.45 × 10^−5^
	rs6432244	2q14.1	12129329	ENSG00000224184	*MIR3681HG*	T/C	38	0.40	0.26	0.63	6.3 × 10^−5^
	rs7571026	2q14.1	15880014	ENSG00000231031	*LINC01804*	A/C	71	0.39	0.24	0.62	7.91 × 10^−5^
	rs1016495	22q11.22	28288136	ENSG00000180957	*PITPNB*	T/C	15	0.31	0.18	0.56	8.84 × 10^−5^
>MPR80	rs2254250	16q12.1	81444857	ENSG00000261609/ ENSG00000153815	*GAN/CMIP*	A/G	27	0.31	0.18	0.53	1.7 × 10^−5^
	rs11240629	1q12	203915958	ENSG00000182004/ ENSG00000237379	*SNRPE/CBX1P3*	A/G,T	43	2.40	1.58	3.65	4.17 × 10^−5^
	rs4669474	2q14.1	5656809	NA	*LOC105373399/ LOC107985842*	T/C	14	5.16	2.31	11.55	6.51 × 10^−5^
	rs7571026	2q14.1	15880014	ENSG00000231031	*LINC01804*	A/C	71	0.39	0.25	0.62	6.57 × 10^−5^
	rs10497702	2q14.1	190345166	ENSG00000115368	*WDR75/ LOC100420666*	T/C	17	0.31	0.17	0.55	6.91 × 10^−5^
	rs2760535	1q11.1	192549912	ENSG00000090104	*RGS1*	G/A	9	0.21	0.10	0.46	7.81 × 10^−5^
	rs2912522	8q13.2	69992380	ENSG00000253658	*RP11-600K15.1*	G/A,T	72	2.66	1.63	4.33	8.42 × 10^−5^
	rs2133127	15q15.1	25137068	ENSG00000128739	*SNRPN*	G/A	33	2.68	1.64	4.38	8.63 × 10^−5^

Abbreviations: Ref/Alt = Reference Allele/Alternative Allele, MAF = Minor allele frequency, OR = Odds ratio, CI = Confidence interval.

**Table 3:** Significant coding variants in PDC80 and MPR80.

	SNP rsID	Chr	Position (hg19)	Major/ Minor Allele	Gene Name	OR	*p*-value	Corrected *p*-Value	AA Change	Gene Location	Effect of AA Change
PDC80	rs9613558	22	28378688	C/G	*TTC28*	0.18	3.46 × 10^−5^	3.11 × 10^−4^	Ala>Pro	Exon 23	Deleterious
	rs7131178	11	93462607	A/T	*KIAA1731*	0.34	3.73 × 10^−4^	3.36 × 10^−3^	Glu>Val	Exon 26	Deleterious
	rs2830585	21	28305212	C/T	*ADAMTS5*	4.02	4.05 × 10^−4^	3.65 × 10^−3^	Arg>His	Exon 5	NA
	rs10888267	1	248059423	C/T	*OR2W3*	2.15	4.19 × 10^−4^	3.77 × 10^−3^	Arg>Cys	Exon 1	Tolerated
	rs4758258	11	7949350	A/G	*OR10A6*	0.40	4.81 × 10^−4^	4.33 × 10^−3^	Leu>Pro	Exon 1	Tolerated
	rs16973457	15	82563991	C/T	*SAXO2*	2.14	4.89 × 10^−4^	4.40 × 10^−3^	Pro>Leu	Exon 2	Tolerated
	rs3795969	2	201354935	C/G	*KCTD18*	2.14	5.16 × 10^−4^	4.64 × 10^−3^	Cys>Ser	Exon 7	Tolerated
	rs2272487	3	126451937	G/T	*CHCHD6*	3.29	5.32 × 10^−4^	4.79 × 10^−3^	Ala>Ser	Exon 4	Deleterious
	rs61741212	19	36157740	C/T	*UPK1A*	3.29	5.96 × 10^−4^	5.36 × 10^−3^	Ala>Val	Exon 1	NA
MPR80	rs3195579	6	54219326	G/A	*TINAG*	0.32	1.43 × 10^−4^	5.70 × 10^−4^	Arg>His	Exon 9	Tolerated
	rs2272487	3	126451937	G/T	*CHCHD6*	3.15	2.18 × 10^−4^	8.70 × 10^−4^	Ala>Ser	Exon 4	Deleterious
	rs7975	14	77793207	G/A	*GSTZ1*	2.54	2.22 × 10^−4^	8.89 × 10^−4^	Glu>Lys	Exon 3	Deleterious
	rs11591349	10	102744331	A/T	*SEMA4G*	2.14	4.72 × 10^−4^	1.89 × 10^−3^	Asp>Val	Exon 14	Deleterious

Abbreviations: Chr = chromosome, OR = odds ratio, AA = amino acid.

**Table 4:** Shows the variance (V) and standard error (SE) for the genetic (G), environmental (e), and phenotypic (p) components of heritability of glaucoma medication adherence for MPR80 and PDC80. The heritability of glaucoma medication non-adherence is represented by the V(G)/V_p_ Variance row.

	MPR80	MPR80 %	PDC80	PDC80 %
V(G) Variance	0.139938	13.99%	0.107453	10.75%
V(G) SE	0.147937	14.79%	0.136762	13.68%
V(e) Variance	0.102986	10.30%	0.115739	11.57%
V(e) SE	0.146327	14.63%	0.135873	13.59%
V_p_ Variance	0.242924	24.29%	0.223192	22.32%
V_p_ SE	0.022742	2.27%	0.02088	2.09%
V(G)/V_p_ Variance	0.576056	57.61%	0.481439	48.14%
V(G)/V_p_ SE	0.603404	60.34%	0.609049	60.90%
*p*-value	0.17064	0.17064	0.2037	0.2037

**Table 5:** Top ten statistically significant (*p* < 0.05) canonical pathways affected by genes identified by IPA analysis for each non-adherence measure.

MPR80		PDC80	
Canonical Pathways	*p*-Value	Canonical Pathways	*p*-Value
GP6 Signaling Pathway	1.25 × 10^−8^	Axonal Guidance Signaling	1.48 × 10^−13^
Insulin Secretion Signaling Pathway	1.25 × 10^−8^	GP6 Signaling Pathway	6.83 × 10^−13^
Synaptogenesis Signaling Pathway	1.63 × 10^−8^	Opioid Signaling Pathway	4.84 × 10^−11^
Opioid Signaling Pathway	3.70 × 10^−8^	Neuropathic Pain Signaling In Dorsal Horn Neurons	8.19 × 10^−11^
Neuropathic Pain Signaling In Dorsal Horn Neurons	8.12 × 10^−8^	Synaptogenesis Signaling Pathway	2.87 × 10^−9^
Dopamine-DARPP32 Feedback in cAMP Signaling	8.53 × 10^−8^	Synaptic Long-Term Potentiation	3.21 × 10^−9^
Semaphorin Neuronal Repulsive Signaling Pathway	1.16 × 10^−7^	Synaptic Long-Term Depression	1.95 × 10^−8^
Axonal Guidance Signaling	1.23 × 10^−7^	Fcγ Receptor-mediated Phagocytosis in Macrophages and Monocytes	2.18 × 10^−8^
Cardiac Hypertrophy Signaling (Enhanced)	1.52 × 10^−7^	Role of NFAT in Cardiac Hypertrophy	4.79 × 10^−8^
Role of NFAT in Cardiac Hypertrophy	4.76 × 10^−7^	Sperm Motility	2.44 × 10^−7^

## Data Availability

The data presented in this study are available on request from the corresponding author. The data are not publicly available due to patient privacy.

## Supplementary Materials

The following supporting information can be downloaded at: https://www.mdpi.com/article/10.3390/ijms24065636/s1.
